# Ectopic Recurrence of Skull Base Chordoma after Proton Therapy

**DOI:** 10.3390/curroncol29040191

**Published:** 2022-03-28

**Authors:** René G. C. Santegoeds, Mohammed Alahmari, Alida A. Postma, Norbert J. Liebsch, Damien Charles Weber, Hamid Mammar, Daniëlle B. P. Eekers, Yasin Temel

**Affiliations:** 1 Department of Radiology and Nuclear Medicine, Maastricht University Medical Center, 6229 HX Maastricht, The Netherlands; l.jacobi@mumc.nl; 2 Department of Radiology, King Fahad Hospital of Imam Abdulrahman Bin Faisal University, Al Khobar 34212, Saudi Arabia; m.alahmari@maastrichtuniversity.nl; 3 Department of Neurosurgery, Maastricht University Medical Center, 6229 HX Maastricht, The Netherlands; y.temel@mumc.nl; 4 School for Mental Health and Neuroscience (MHeNS), Maastricht University Medical Center, 6229 HX Maastricht, The Netherlands; 5 Department of Radiation Oncology, Massachusetts General Hospital, Boston, MA 02114, USA; nliebsch@mgh.harvard.edu; 6 Center for Proton Therapy, Paul Scherrer Institut (PSI), 5232 Villigen, Switzerland; damien.weber@psi.ch; 7 Curie Institute, Department of Radiation Oncology, CPO, 26, Rue d’Ulm, 75005 Paris, France; hamid.mammar@curie.fr; 8 Department of Radiation Oncology (Maastro), GROW School for Oncology, Maastricht University Medical Centre, 6229 HX Maastricht, The Netherlands; danielle.eekers@maastro.nl

**Keywords:** chordoma, skull base, proton therapy, recurrence, surgery

## Abstract

**Simple Summary:**

Chordoma are very rare tumors of the spine and skull base. Due to close proximity of crucial organs, like the brain stem, complete removal can often not be achieved, and tumor tissue, either macroscopic or microscopic, remains in situ. Local recurrence up to 88% occurs in 10 years. Ectopic recurrence as an early sign of treatment failure is considered rare. We retrospectively reviewed five patients with ectopic recurrence as a first sign of treatment failure after treatment with surgery and proton therapy, and studied the applied treatment strategies and imaging follow-up. We found 18 ectopic recurrences in these five patients, of which 17 (94%) could be related to prior surgical tracts. Our theory is that these relapses occur due to microscopic tumor spill during surgery. These cells did not receive a therapeutic radiation dose. Advances in surgical possibilities and adjusted radiotherapy target volumes might improve local control and survival.

**Abstract:**

Background: Chordoma are rare tumors of the axial skeleton. The treatment gold standard is surgery, followed by particle radiotherapy. Total resection is usually not achievable in skull base chordoma (SBC) and high recurrence rates are reported. Ectopic recurrence as a first sign of treatment failure is considered rare. Favorable sites of these ectopic recurrences remain unknown. Methods: Five out of 16 SBC patients treated with proton therapy and surgical resection developed ectopic recurrence as a first sign of treatment failure were critically analyzed regarding prior surgery, radiotherapy, and recurrences at follow-up imaging. Results: Eighteen recurrences were defined in five patients. A total of 31 surgeries were performed for primary tumors and recurrences. Seventeen out of eighteen (94%) ectopic recurrences could be related to prior surgical tracts, outside the therapeutic radiation dose. Follow-up imaging showed that tumor recurrence was difficult to distinguish from radiation necrosis and anatomical changes due to surgery. Conclusions: In our cohort, we found uncommon ectopic recurrences in the surgical tract. Our theory is that these recurrences are due to microscopic tumor spill during surgery. These cells did not receive a therapeutic radiation dose. Advances in surgical possibilities and adjusted radiotherapy target volumes might improve local control and survival.

## 1. Introduction

Chordomas are rare tumors of the axial skeleton that occur most frequently at the sacrum and base of the skull [1]. It is assumed that chordoma arise from notochordal remnants that remain in the axial skeleton during life, and may become malignant at any age [2]. The incidence is estimated around 0.05 to 0.08 per 100,000 per year [1,3]. The 10-year overall survival rate of skull base chordoma is around 55% [4]. Curation is difficult, and has a high local recurrent rate of 53% at 5 years and 88% at 10 years [5]. The tumor is preferably maximally resected followed by postoperative irradiation of the resection cavity, including a margin around the (pre-operative) tumor bed. However, maximal resection is often not a complete resection at the skull base due to the close relation to crucial and sensitive neural and vascular structures, like brain stem, optic nerve/chiasm, and carotid and basilar arteries [6]. Consequently, tumor tissue often remains in situ, either macroscopic or microscopic. As chordoma are resistant to standard dose radiotherapy [7], the tumor bed is irradiated by high dose radiotherapy (>65 GyRBE). To minimize the dose to the surrounding organs at risk (OARs), the dose is preferably delivered in the form of particle therapy [8]. Of the patients treated with proton therapy after surgery, 26% still develop recurrence [9], and local recurrence is the predominant form of treatment failure [10]. Recurrences are also described in the vicinity, but outside of the primary tumor bed, as well as at a distance [11,12]. Ectopic recurrences are defined as recurrences outside of the primary tumor bed. Ectopic recurrences in the vicinity of the primary tumor are considered rare, especially in the early stage of the disease [11]. It remains unclear why chordoma only in selected patients show ectopic recurrence, and favorable sites of ectopic recurrences in the skull base chordoma are unknown. The primary goal of this study is to find a pattern in the ectopic recurrences in the skull base chordoma. Knowing the preferable sites of recurrence may influence treatment strategies, aid in early detection during follow up imaging, and may improve outcomes in patients. Because chordoma recurrences are difficult to distinguish from postoperative distortions in anatomy and radionecrosis, the secondary goal of this study is to study the imaging parameters of recurrences and to identify possible pitfalls during follow-up. In this study, we describe five patients with ectopic recurrence as a first sign of treatment failure and correlate these recurrences with previous treatment strategies and imaging follow-up.

## 2. Materials and Methods

### 2.1. Medical Ethics Committee Approval

This retrospective study was approved by the medical ethics committee of the Maastricht University Medical Center (MUMC+), reference number METC2018-0740. Research was conducted in accordance with the declaration of Helsinki. All patient data were anonymized and coded. Patients who objected to evaluating their medical data were excluded from the study. The study was submitted to trialregister.nl with reference number NL7821. All patients were referred to specialized proton therapy centers, namely Massachusetts-General Hospital (Boston, MA, USA), Paul-Scherrer Institut (PSI, Villigen, Switzerland), and Institut Curie (Paris, France). Isodose information and treatment plans were requested from these centers. The study was also approved by the institutional review board of Maastro Clinic in Maastricht, reference number W 19 10 00055.

### 2.2. Patient Selection Process

The radiological and surgical reports between 1999 and 2019 in the database of MUMC+ were searched for the term “chordoma” in order to identify all chordoma patients that were treated at our center. Patients with chordoma of the skull base who did not have prior treatment were manually selected. The eligibility criteria were as follows: (1) histologically proven chordoma without previous treatment, (2) location at the base of the skull or cranio−cervical junction, (3) treated with surgery and postoperative proton therapy, and (4) development of recurrence outside of the primary tumor bed during follow-up as a first sign of treatment failure.

### 2.3. Imaging and Recurrence Detection

The standard imaging follow-up was performed by magnetic resonance imaging (MRI) of the cerebrum and/or cervical spine with T2-weighted images, T1-weighted images pre- and post-gadolinium contrast, diffusion weighted imaging (DWI) with ADC mapping and FLAIR for the cerebrum and sagittal, and axial T2 and T1 pre- and postcontrast for the cervical spine. In some cases, T2 STIR was added in the axial and/or coronal plane. Two patients were treated for a chordoma at the cranio−cervical junction. These patients did not initially undergo a routine follow-up MRI of the whole cervical spine. Imaging of the cranio−cervical junction in these patients was performed by the cerebral MRI protocol.

MRI scans performed during the follow-up period were re-evaluated to define all recurrences separately. Recurrence was diagnosed when new lesions appeared that were radiologically suspicious for recurrence. Of all included patients, at least one recurrence was histologically proven chordoma. Recurrence was evaluated on T2-weighted images and T1-weighted images pre- and post-contrast. In most cases, diffusion weighted imaging was used as well. Restricted diffusion was visually analyzed and defined as high signal intensity on high-B value images with a low intensity on apparent diffusion coefficient (ADC) images. Each lesion per patient was given a number, sequential in time of appearance. Two or more lesions that were identified at the same time were characterized as separate lesions if there was no visible connection between the two, and the lesion was clearly in a different location to the earlier defined lesions. However, at the end-stage of the disease, multiple (>5) lesions were sometimes visible. These were defined as one group of recurrences per anatomic location. For every defined lesion, the radiological reports associated with the imaging studies were evaluated and checked when a lesion was first described, and were compared to when a lesion was visible first, to calculate the possible diagnostic delay. It should be noted that the possible diagnostic delay was calculated as the time interval between the imaging study in which a recurrence was described first and the date of the imaging study in which the slightest signal or anatomical change in this area was visible. The signal characteristics of the recurrences were analyzed compared to the original tumor, in particular on diffusion weighted imaging and contrast enhancement.

### 2.4. Treatment Evaluation

#### 2.4.1. Surgery

The surgical reports were analyzed for the approach, goals of the surgery, and outcome (total macroscopic resection versus macroscopic remnant). The time interval between surgery and radiotherapy was studied, as well as the number of surgeries before the detection of the first ectopic recurrence. Moreover, surgical interventions that were a result of complications due to prior surgery or stabilization of the cranio−cervical junction were also studied.

#### 2.4.2. Proton Therapy

**Figure 1:**
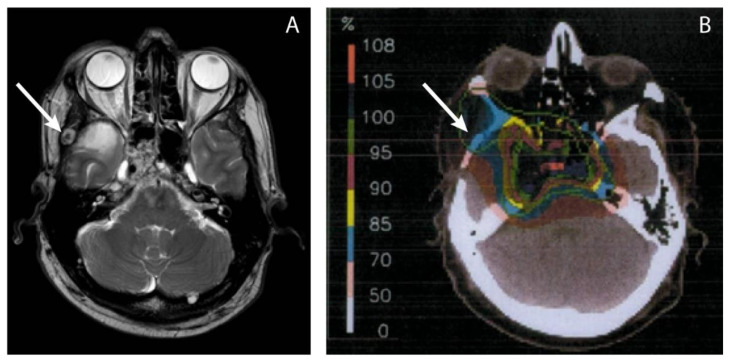
The delivered dose in the area where a metastasis was later found, was visually estimated using MRI images of the ectopic recurrence (**A**) and the radiotherapy dose plans (**B**). The arrow points toward the recurrence (**A**) and the estimated location on the dose plan (**B**), which is in the 70% isodose area, according to the isodose scale. With the prescribed dose of 100% being 74 GyRBE, the estimated dose to the region of local recurrence was 0.7 × 74 GyRBE = 51.8 GyRBE.

All defined lesions were separately correlated with the proton therapy plans. The radiotherapy reports were checked to ensure the delivered dose to the target volume. The dose to the ectopic recurrence not visible at the time of proton therapy was retrospectively determined, in good cooperation with the treating proton centers. The location of recurrences on the MRI were linked with the initial dose plan by visual estimation in order to estimate the dose. An example of this visual estimation is illustrated in Figure 1.

## 3. Results

### 3.1. Patient Cohort Characteristics

The search of the surgical and radiological database with manual selection of the skull base chordoma without prior treatment revealed 30 histologically proven skull base chordoma patients. Of all of the patients, 62% (*n* = 16) received proton therapy, of which five patients (32%) had ectopic recurrence as a first sign of treatment failure. Out of these five patients, one patient had local recurrence at the same time as the first ectopic recurrence and one patient developed local recurrence during later follow-up. No metastasis at distance were found during follow-up.

### 3.2. Follow-Up

**Figure 2:**
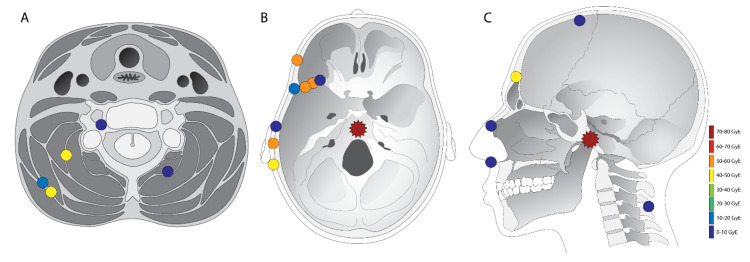
Overview of all defined chordoma lesions. The primary tumor is marked with a star, and ectopic recurrences with a dot. The colour of the markings are an estimation of the dose in GyRBE that was delivered at the site where later the recurrence was visible. All lesions are only marked once in either (**A**,**B**) or (**C**). The location in the figure is an approximation of the actual recurrence location, so as to fit all the lesions in these three illustrations. However, the compartment of the lesion (subcutaneous, fibrous tissue/muscle, or bone) is accurate.

**Figure 3:**
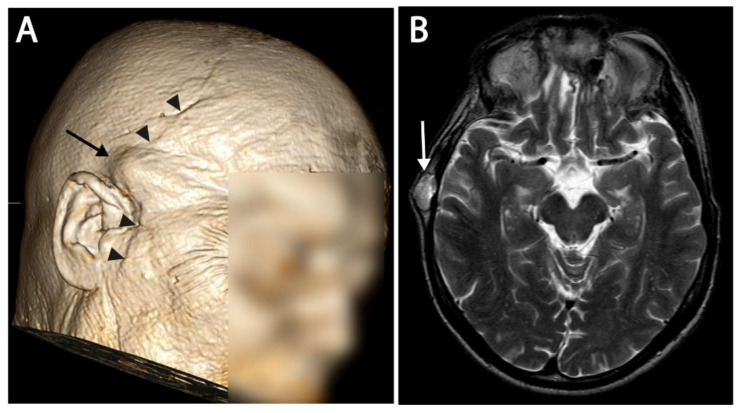
(**A**) 3D reconstructed image of an MRI. A subcutaneous recurrence is visible as a bump under the skin, and is indicated with an arrow. The surgical scar from the coronal midfacial approach is also visible and marked with arrowheads. (**B**) T2 image of the same patient at the level of recurrence. Subcutaneous recurrence is marked with an arrow.

During follow-up, a total of 18 ectopic recurrences were defined in five patients. An overview of the lesion locations is illustrated in Figure 2. Comparing the recurrences to the prior surgical approaches, 17 out of 18 (94%) ectopic recurrences could be related to the surgical tract. Recurrences were found in or under the cutaneous incision, in dissected muscle and fibrous tissue, or in the drilled bone. An example of subcutaneous recurrence is demonstrated in Figure 3. One patient had recurrence at the neuroforamen a few levels below the primary tumor, which could be regarded as drop metastasis via cerebrospinal fluid or hematogenous metastasis via the venous plexus of the vertebral artery. Of all 18 ectopic recurrences, the center of the lesion could be found in the bone in five cases, in muscle/fibrous tissue in six cases, and subcutaneous in seven cases. All patients who developed recurrences, eventually developed recurrences on multiple sites. Recurrences were T2 hyperintense in 94%, T2 isointense in 6%, showed contrast enhancement in 72%, and restricted diffusion in 36%. Comparing the first description of a lesion in the radiological report to the first visible signal intensity or anatomical change in this area on MRI in retrospect, diagnostic delay was on average 76 days (0–449 days). The first diagnosed ectopic recurrence in all patients had a diagnostic delay (34–449 days).

### 3.3. Surgery

A total of 31 surgical interventions were performed on five patients between 2003 and 2016, with a median of 6 (range 3–11) per patient. The first surgery was performed at an average of 41 days (4–87 days) after diagnosis. A median of three surgeries (range 2–4) per patient were performed before the start of radiotherapy. This was because of multiple reasons. In one patient, multiple different approaches were necessary in order to resect as much tissue as possible. In one patient, postoperative MRI revealed remnant tumor tissue at the site, after which new surgery was planned two days later. Three out of five patients did not receive immediate radiotherapy after resection and had regrowth of the tumor remnant at the primary site. After re-resection, additional proton therapy was applied in these patients. Macroscopic complete resection of the primary tumor was achieved in 27% of cases. Complete macroscopic removal was reported in 50% of all resections, including resection of ectopic recurrences. In most cases, the goal of surgery was to remove as much tumor tissue as possible. In one case, the goal was to remove additional tumor mass to facilitate radiation therapy. Five non-oncologic surgeries were performed. Two patients underwent occipito−cervical fusion to stabilize the cranio−cervical junction after the primary tumor resection via the far lateral approach. Two patients experienced recurrent sinonasal infections due to prior surgery and radiotherapy, one of which had to undergo an infundibulotomy twice. The same patient also experienced recurrent otitis media and had to undergo middle ear drainage as well.

One surgery was performed due to possible tumor regrowth, which was not clearly distinguishable from the radiation necrosis. After inspection of the anterior temporal lobe area, no mass was found and the imaging findings were considered to be radiation necrosis. An overview table of the surgical approaches and treatment of recurrences is summarized in Table 1.

### 3.4. Radiotherapy

All five patients in this study received adjuvant proton therapy at a single site with an average dose of 74 GyRBE (68–78 GyRBE). The dose was delivered in 1.8 to 2 GyRBE per fraction. In two cases, a combination of proton and photon therapy was utilized. In these cases, 14–20 Gy was delivered via intensity-modulated radiation therapy (IMRT) in 7–10 fractions. In the other three patients, the complete dose was delivered via protons. "All centers used their own “standard chordoma proton therapy protocol”, with no significant concession needed because of dose restraints due to the proximity of OARs (i.e., cochlea, brain stem, optic nerve, and optic chiasm). The goal of radiation therapy was to improve locoregional control and overall survival in all cases. The planned target volume was the volume of the primary tumor before surgery, including a marge with or without remnant tumor tissue. Details of the delivered proton therapy are shown in Table 2.

In addition to proton therapy to the primary tumor, other types of radiotherapy were utilized in the treatment of the regrowth of the primary tumor and ectopic recurrences. The goal of this treatment was to delay tumor progression. Proton therapy was applied a second time in one patient, and three patients were treated with 3D conformal or IMRT photon therapy.

## 4. Discussion

The primary goal of this study was to identify a possible pattern of recurrence in skull base chordoma patients after treatment with surgery and proton therapy. This study describes five patients who developed ectopic recurrence as a first sign of treatment failure after treatment with surgery and proton therapy identified in our institutional database. It was unexpected that 94% of the ectopic recurrences were related to the surgical tract, with no preferable tissue type (subcutis, bone, muscle, or fibrous tissue) for ectopic recurrence. Even though recurrences in the surgical tract in skull base chordoma have been described in the literature as case reports [13,14,15,16,17,18,19] and systematic reviews [12,20], it remains under-evaluated. In our hospital, a total of 16 skull base chordoma patients received proton therapy after surgery, and five patients (32%) showed ectopic recurrence. This high occurrence of ectopic recurrence may partially be explained by the relatively small subset of patients. However, surgical tract recurrence is only described in the literature in 1.4 to 7.3% of cases [11,18,21,22,23], which is substantially lower than in our cohort. This difference may be due to the fact that there is no clear definition of surgical tract recurrence. In our study, 17/18 ectopic recurrences could be related to the surgical incision and route of the primary tumor resection, and had a clear distance between the recurrence and primary tumor. One recurrence was considered, a drop metastasis with epidural spread via the cervical spine in the cranio−cervical junction surgical route. It was not ruled out that this recurrence was due to lymphatic or hematogenic spread via the venous plexus of the vertebral artery. As chordomas metastasize to almost all tissue types with no clear predilection for bone, soft tissue, or (sub)cutis, some other ectopic recurrences in our study could have been due to lymphatic or hematologic tumor spread as well. However, no metastasis at distance were seen in our study population. Multiple ectopic recurrences in the vicinity of the primary tumor without distant metastasis did not correlate well with hematologic tumor spread.

Vogin et al. studied 371 patients with chordoma, and found ectopic relapses in 13 patients, which was 1.4% of the cases. However, subcutaneous recurrence was not regarded as surgical tract recurrence, which we did in our study. Some subcutaneous recurrences were not directly under the surgical scar. These recurrences were still considered in the surgical tract, as during surgery, the cutis was lifted and retracted, and therefore the surgical tract at the cutis was considerably larger than the postoperative changes visible on the MRI. Another explanation of the higher incidence in our study may be the difference in surgical technique between our population and the literature. Fernandes et al. [24] described two cases with presumed iatrogenic seeding after endoscopic endonasal surgery (EES). The surgical access of this technique is much smaller than in open surgery, which may reduce the risk of iatrogenic tumor seeding.

In 2001, Arnautovic et al. [21] described the impact of tumor seeding in the surgical cavity, and even at distance in the abdominal wall where fat was harvested for facial reconstruction. These authors suggest coating the walls of the operative tunnel with fibrin glue and large cotton patties, and all instruments that came in contact with the tumor should be considered to be contaminated. Unfortunately, there are no follow-up data available for this surgical technique with regard to surgical cavity seeding.

One explanation for the high recurrence rate in the surgical pathway is that high viscous matrix produced by chordoma-cells may promote the adhesion of the tumor cells to the surgical instruments. To our knowledge, no studies have been performed to check the surgical instruments for remnants of chordoma tissue or cells. One patient developed recurrences in the surgical cavity despite irradiation of up to 54 Gy. This confirms that chordoma are resistant to radiotherapy if the dose is not high enough [7,25]. This triggers the question whether the surgical cavity should receive a therapeutic dose as well. To the best of our knowledge, no previous study has reported radiation of the surgical cavity with a dose higher than 65 Gy. Moreover, irradiating the surgical cavity may result in severe adverse events. Close follow-up of the surgical cavity may be more beneficial to chordoma patients. When recurrences in the surgical cavity appear, treating the whole surgical cavity could be considered.

**Figure 4:**
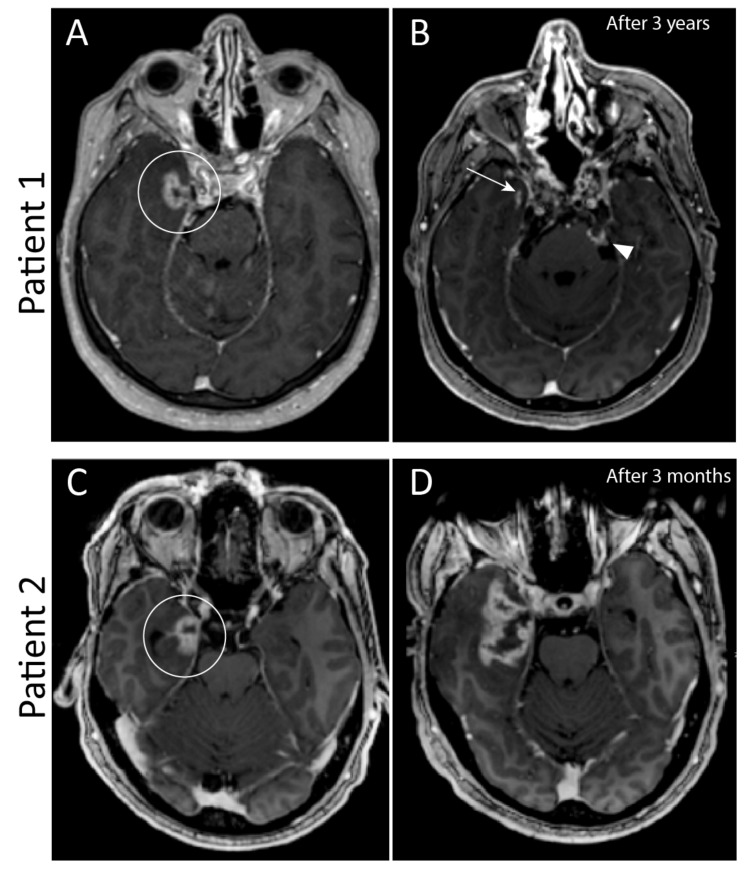
Two patients with similar contrast enhancement of the medial temporal lobe three years after radiotherapy in patient 1, and one year after radiotherapy in patient 2 (circled in (**A**,**C**)). This contrast enhancement is consistent with either radiation necrosis or tumor progression. In (**A**) there are also hyperintense foci in the upper cerebellum, which are due to phase encoded pulsation artefacts of the carotid artery on both sides and not a true enhancement. Follow-up imaging after three months showed no differences in patient 1, and imaging after 3 years (**B**) showed clear regression of enhancement of the temporal lobe (arrow). However, this patient had tumor progression in the clivus and cerebellopontine cistern on the left side (arrowhead). In patient 2, follow-up imaging after three months (**D**) showed clear progression in enhancement and mass effect. Tissue biopsy confirmed that this was tumor progression.

The secondary goal of this study was to study the imaging parameters of recurrences and to identify possible pitfalls in the follow-up imaging of the skull base chordoma. Radiation necrosis is common in chordoma treated with particle therapy [9]. Radiation necrosis has similar imaging features as tumor progression, and typically appears on MRI as contrast-enhanced areas with a central necrotic core, surrounded by hyperintensity on T2/FLAIR due to vasogenic oedema [26]. Radiation necrosis typically does not show restricted diffusion, with intermediate to high signal intensity on high b-value DWI and high signal intensity on the corresponding ADC map, a phenomenon known as T2 shine through [27]. Up to 36% of the primary tumors/recurrences in our study group showed diffusion restriction on MRI, which makes diffusion restriction in a lesion suspicious for recurrence, but lack of diffusion restriction does not confirm radiation necrosis. Figure 4 illustrates the challenges of differentiating radiation necrosis from tumor progression.

The biggest weakness of this study was the small cohort, which hindered solid conclusions and may explain the relatively high ectopic recurrence rate. Prospective analysis of larger cohorts is necessary to confirm the occurrence of ectopic recurrence as a first treatment failure. Another weakness of this study was that only patients treated with proton therapy were evaluated. A quick evaluation of the other patients treated at our hospital with either no adjuvant radiotherapy or photon radiotherapy showed that ectopic recurrences in the surgical pathway indeed occurred in this patient group. This is not surprising as the surgical pathway is usually not irradiated with a therapeutic dose.

A strength of this study was that all patients’ imaging exams and treatment plans were thoroughly re-evaluated. All ectopic recurrences were studied in detail and the smallest changes in anatomy in locations where later recurrences were found were analyzed to improve follow-up strategies and prevent therapeutic delay.

This study describes five patients who developed ectopic recurrent disease as a first sign of treatment failure after the current best treatment strategy—namely surgery with adjuvant high-dose radiotherapy. The question remains in what way this treatment strategy could be improved in the future. In our series, multiple surgical approaches were necessary to remove as much tumor tissue as possible, and to facilitate proton therapy. We suggest that the risk of tumor spread during surgery should be taken into consideration and limitation of the number of surgeries and approaches could be considered. Early detection of ectopic recurrences is difficult but important in the follow-up of chordoma and for the treatment of side effects. Table 3 shows some imaging features in follow-up that we experienced to be relevant for the early detection of ectopic recurrence.

Considering the complexity of therapy, while surgical excision with a margin is not possible and the proximity of organs at risk limits radiotherapy, it seems useful to discuss the complete treatment plan in a multidisciplinary team after diagnosis, including the management of recurrence of the primary chordoma and treatment for ectopic recurrences. This may improve the local control and mortality of chordoma patients in the future.

## 5. Conclusions

In this study, we found that ectopic recurrences of chordoma occur in the surgical tract, which could be more common than previously thought. However, our cohort is relatively small and more research in larger cohorts is needed to support this finding. Our theory is that these recurrences are due to microscopic tumor spill during surgery. These cells did not receive a therapeutic radiation dose. Improvements in surgical techniques and radiotherapy target definition might improve (recurrence-free) survival. Knowledge of and focus on subtle differences in the surgical tract during radiologic follow-up may detect recurrent disease at an earlier stage and may improve locoregional control.

## Figures and Tables

**Table 1:** Surgical approaches and location of ectopic recurrences. The cutaneous approach was used in subcutaneous recurrence, meaning that the skin was incised superficially to the tumor tissue. Linac = linear accelerator. GK = gamma knife. RT = radiotherapy.

Patient Number	Primary Tumour Location	Surgical Approaches	Postoperative Radiotherapy	Ectopic Recurrence Site	Time to First Ectopic Recurrence (Months)	Treatment of Recurrence
1	Clivus	○ *Before RT*: 2x Pteryonal right ○ *Salvage*: ○ 2x Pteryonal right ○ 3x Cutaneous	Proton	2x Ala major os sphenoid 2x Cutaneous nodules	9	Surgery Photon radiotherapy (Linac)
2	Craniocervical junction	○ *Before RT*: 3x Far lateral right ○ *Salvage*: ○ Cutaneous ○ Anterolateral cervical ○ *Other*: ○ Occipitocervical fusion	Proton/Photon	Paravertebral paramedian right Neuroforamen C4-C5 right	7	Surgery Proton therapy
3	Craniocervical junction	○ *Before RT*: Far lateral left & right ○ *Salvage*: 3x Cutaneous ○ *Other*: Occipitocervical fusion	Proton	Subcutaneous retroauricular Subcutaneous neck 3x Neck muscle left + right	18	Surgery Photon radiotherapy (Linac)
4	Clivus	○ *Before RT*: 2x Transnasal ○ *Salvage*: Transnasal	Proton	Nose septum Subcutaneous nose	147	Surgery
5	Clivus	○ *Before RT*: ○ Transnasal ○ Facial degloving ○ *Salvage*: ○ 4x Cutaneous ○ 2x Subfrontal ○ *Other*: ○ 2x Infundibulotomy ○ Middle ear drainage	Proton / Photon	Ala minor os sphenoid Subcutaneous preauricular Frontal bone Subcutaneous frontal Dura mater frontal right	41	Surgery Photon radiotherapy (Linac)

**Table 2:** Radiotherapy data and timing of radiotherapy compared to surgery and development of ectopic recurrence. The first recurrence area radiotherapy dose is the dose that was delivered to the area where the first diagnosed recurrence appeared. RT = radiotherapy. GyRBE = Gray equivalent.

Patient Number	Primary Tumour Dose (GyRBE)	Number of Fractions	Protocol	Regrowth of Primary Tumour after RT (Months)	Number of Ectopic Recurrences	First Ectopic Recurrence Area RT Dose (GyRBE)	Time Interval between Radiotherapy and First Ectopic Recurrence (Months)	Time Interval between First Surgery and RT (Months)
1	74	37	54 GyRBE + 20 GyRBE boost	9	4	54	9	5
2	78	39	56 GyRBE proton + 20 Gy photon boost	No regrowth of primary tumor	2	1	22	18
3	74	41	Standard skull base chordoma protocol	37	5	50	24	24
4	74	37	54 GyRBE + 20 GyRBE boost	No regrowth of primary tumor	2	1	98	23
5	68	34	48 GyRBE proton + 20 Gy photon Boost	No regrowth of primary tumor	5	1	43	5
Average	74	37.6		39	3	21	41	17

**Table 3:** Imaging of chordoma: Lessons learned.

All prior surgical pathways should be examined during follow up. In cases of craniocervical approaches, like far lateral approach, the cervical spine should be imaged as well.
Reporting of all surgical pathways may aid in early detection of recurrence.
The most important follow-up MRI sequence is T2, as only 72% of the recurrences show contrast enhancement. Fat suppression sequences can aid in early detection of small subcutaneous recurrence, to differentiate from fat tissue.
DWI is (only) useful in follow-up if other chordoma lesions in the same patient show restricted diffusion as well.
Radiation necrosis can be difficult to differentiate from tumour recurrence. Close follow-up, comparison with prior lesions and radiation field is necessary.

## Data Availability

The authors confirm that the data supporting the findings of this study are available within the article.
